# Sensitive and Selective Electrochemical Detection of Hydrogen Peroxide Using a Silver-Incorporated CeO_2_/Ag_2_O Nanocomposite

**DOI:** 10.3390/bios15090617

**Published:** 2025-09-17

**Authors:** Gunasekaran Manibalan, Govindhasamy Murugadoss, Dharmalingam Krishnamoorthy, Venkataraman Dharuman, Shaik Gouse Peera

**Affiliations:** 1 Department of Chemistry, Indian Institute of Technology Bombay, Mumbai 400076, Maharashtra, India; gmanibalanphys@gmail.com; 2 Centre for Nanoscience and Nanotechnology, Sathyabama Institute of Science and Technology, Chennai 600119, Tamil Nadu, India; 3 Department of Physics, Kongunadu College of Engineering and Technology, Tholurpatti, Trichy 621215, Tamil Nadu, India; kmbarani@gmail.com; 4 Molecular Electronics Laboratory, Department of Bioelectronics and Biosensors, Science Campus, Alagappa University, Karaikudi 630003, Tamil Nadu, India; dharumanv@alagappauniversity.ac.in; 5 Natural Science Research Institute, College of Natural Sciences, Keimyung University, 1095 Dalgubeol-daero, Daegu 42601, Republic of Korea

**Keywords:** Ag-doped CeO_2_/Ag_2_O catalyst, chemical route, hydrogen peroxide, sensitivity, sensor

## Abstract

Precision and real-time detection of hydrogen peroxide (H_2_O_2_) are essential in pharmaceutical, industrial, and defence sectors due to its strong oxidizing nature. In this study, silver (Ag)-doped CeO_2_/Ag_2_O-modified glassy carbon electrode (Ag-CeO_2_/Ag_2_O/GCE) has been developed as a non-enzymatic electrochemical sensor for the sensitive and selective detection of H_2_O_2_. The synthesized Ag-doped CeO_2_/Ag_2_O nanocomposite was characterized using various advanced techniques, including X-ray diffraction (XRD), Fourier-transform infrared spectroscopy (FT-IR), field-emission scanning electron microscopy (FE-SEM), and high-resolution transmission electron microscopy (HR-TEM). Their optical, magnetic, thermal, and chemical properties were further analyzed using UV–vis spectroscopy, electron paramagnetic resonance (EPR), thermogravimetric-differential thermal analysis (TG-DTA), and X-ray photoelectron spectroscopy (XPS). Electrochemical sensing performance was evaluated using cyclic voltammetry and amperometry. The Ag-CeO_2_/Ag_2_O/GCE exhibited superior electrocatalytic activity for H_2_O_2_, attributed to the increased number of active sites and enhanced electron transfer. The sensor displayed a high sensitivity of 2.728 µA cm^−2^ µM^−1^, significantly outperforming the undoped CeO_2_/GCE (0.0404 µA cm^−2^ µM^−1^). The limit of detection (LOD) and limit of quantification (LOQ) were found to be 6.34 µM and 21.1 µM, respectively, within a broad linear detection range of 1 × 10^−8^ to 0.5 × 10^−3^ M. The sensor also demonstrated excellent selectivity with minimal interference from common analytes, along with outstanding storage stability, reproducibility, and repeatability. Owing to these attributes, the Ag-CeO_2_/Ag_2_O/GCE sensor proved effective for real sample analysis, showcasing its potential as a reliable, non-enzymatic platform for H_2_O_2_ detection.

## 1. Introduction

In recent times, due to the ontogenesis response of biological sensors, they have garnered extensive research attention in diverse fields, such as nanotechnology and materials science [1]. An enormous effort is made to create and characterize inorganic materials for cutting-edge bioelectrodes that have a noticeable shape and show physicochemical properties when interacting with biomolecules and other bio-recognition event derivatives. In a row with the above properties, the improvement of efficient catalysts plays an essential role, which is utilized for water oxidation, dye degradation, water splitting, pollutant removal, and sensor applications [2,3,4]. Numerous methods have been used for many years to detect H_2_O_2_, including titration, chromatography, light detection, and electrochemical sensors. On the other hand, electrochemical sensors measure changes in chemical energy using an electrical transducer. When there is no current, potentiometric sensors calculate the potential (voltage) between the probes. In the case of amperometry sensors, the current is measured while the potential (voltage) is maintained. Among them, the Electrochemical technique is a successful method that is extensively used in biosensor experiments by virtue of its immense sensitivity, swift response, and facile and reliable calculation of definite analytes [5]. The analytical diagnosis of hydrogen peroxide plays a significant role in the environmental, industrial, and pharmaceutical fields. It also holds promise for use in the water sector, including as a bleaching agent in milling industries, municipal water treatment, drinking water treatment, and as a treatment for gas, oil, and petrochemical refineries, as well as in the manufacturing of polymer products [6]. The electrochemical sensor can be made highly efficient by choosing an appropriate electrode material as the working electrode for the determination of hydrogen peroxide [7].

For the detection of a specific analyte using the electrochemical methods, various metal oxide nanocatalysts such as TiO_2_ [8], CeO_2_ [9], ZnO [10], MO [11], CuO [12], FeO [13], MnO_2_ [14], NiO [15], Fe_2_O_3_ [16], SnO_2_ [17], V_2_O_5_ [18], and MoO_3_ [19] were used. The nanocatalyst shows remarkable physical and chemical properties that play a crucial role in the determination of the various chemical and electrochemical reactions [20]. Because of their excellent electrical conductivity, metal oxides such as CeO_2_ and Ag_2_O nanostructures established using modified electrodes demonstrate H_2_O_2_ sensors that have good sensitivity and reduced interference from other oxidizable species [5,21]. Similarly, the rare-earth metal oxide, cerium oxide (CeO_2_), has received huge research attention as a catalyst for electrochemical sensor applications, as well as bioassays and antioxidant therapy [22,23]. The enhanced catalytic performance in various fields is governed by the existence of diverse valence states of Ce^3+^ and Ce^4+^ followed by the existence of oxygen vacancies. The primary factor that operates the catalytic performance is the possibility of switching of redox couple between each state CeO_2_ ↔ CeO_2_−x + x/_2_O_2_ (Ce^4+^ ↔ Ce^3+^), which is a recycle process [24,25]. In essence, the surface of oxygen is where the catalytic activity begins, and an improvement in the surface oxygen will result in an improvement in the catalytic performance [7]. They also possess some interesting parameters like biocompatible, high iso electric point, high ionic conductivity [5], and offer a greater number of active sites [26]. Though CeO_2_ shows attracting features, it behaves as a poor electrical conductor due to its wide band gap [27]. Moreover, single metal oxides or metals have some limitations, such as low electron transfer efficiency and fewer oxygen vacancies. Thus, doping semiconductor material with CeO_2_ possesses numerous oxidation states and high electron transfer efficiency, which is favourable for improving electrocatalytic activity for the electrochemical sensing performances [28]. To improve the conductivity and active side of the CeO_2_, it can be modified by different methods, like doping suitable metal ions or adding a low band gap material with CeO_2_ as a nanocomposite. Modifying the CeO_2_ by doping a metal ion is an attractive route for electrochemical sensor applications. Various transition metal nanoparticles were doped with CeO_2_ to modify the optical and electrical properties. Among the familiar nanoparticles, silver (Ag) is suitable for its favourable optical, electrical, and photothermal properties [28]. Further, Ag nanoparticles are characterized by better chemical stability and good catalytic activity. Additionally, Ag is one of the potential candidates for conventional electrocatalysts due to its better electron transfer ability [29]. The incorporation of CeO_2_ nanoparticles as a catalyst can enhance the electrocatalytic property. For the first time, an Ag-doped CeO_2_/Ag_2_O-modified electrode was examined for the electrochemical sensing of H_2_O_2_ analyte. The electrocatalytic activity of Ag-CeO_2_/Ag_2_O/GCE reveals the high sensitivity, excellent selectivity, better reproducibility, repeatability, and long-term stability. Moreover, the actual sample analysis demonstrates good recovery for the detection of H_2_O_2_.

## 2. Materials and Methods

### 2.1. Materials

We purchased 99.9% pure cerium nitrite and silver AgNO_3_ and Ce(NO_3_)_3_. 6H_2_O from Sigma-Aldrich in Bangalore, India. Polyvinylpyrrolidone (PVP, MW 40,000), hydrogen peroxide (H_2_O_2_, 99.9%), sodium hydroxide, acetone, ethanol, sodium phosphate dibasic (Na_2_HPO_4_, 99.9%), sodium phosphate monobasic (NaH_2_PO_4_, 99%), ascorbic acid, uric acid, dopamine, and glucose were provided by Sisco Research Laboratories Pvt. Ltd. (SRL) in India.

### 2.2. Silver-Doped CeO_2_/Ag_2_O Nanocomposite Synthesis

A facile chemical co-precipitation method was used to synthesize the Ag-CeO_2_/Ag_2_O nanocomposite. After dissolving 0.1 M of Ce(NO_3_)_3_·6H_2_O in 50 mL of de-ionized water, 0.5 g of PVP was added to the mixture. Subsequently, the solution mentioned above was mixed with 0.1 M AgNO_3_ in 50 mL of de-ionized water. Then, 0.3 M NaOH in 50 mL de-ionized water was gradually added to the colloidal solution, and the mixture was stirred for the next two hours. Eventually, the wet sample was washed several times with deionized water, acetone, and ethanol before being kept at 160 °C for 12 h in an oven. Finally, the Ag-doped CeO_2_/Ag_2_O nanocomposite was collected for additional examination.

### 2.3. Material Characterization

A PW 3040/60 X-ray diffractometer equipped with a Cu-K source (radiation at 1.54 A) was used to examine the size and crystal structure of the nanoparticles. The surface microstructural and elemental composition of the sample was studied using field-emission scanning electron microscopy (FE-SEM) (Apreo 2S HiVac; Thermofisher Scientific, Waltham, MA, USA) and EDS elemental mapping analysis (Ultim Max 40, Oxford Instruments, Abingdon, UK). Using an FEI-Tecanai 20 G2 transmission electron microscope, the morphology of the synthesized samples was examined. The FT-IR (BRUKER-TENSOR 27) spectrometer was used to analyze the functional groups of the nanoparticles using KBr pellets. The samples’ optical characteristics were inspected using a (VARIAN Cary 500 Scan) UV-Visible spectrometer. The magnetic properties of the samples were studied by electron paramagnetic resonance (EPR) using a BRUKER BIOSPIN, EMX Plus EPR analyzer. The thermal analysis was inspected using TG-DTA (SDT, Q600 thermal analyser with 10°/min at room temperature to 1000 °C under N_2_ gas conditions. The chemical composition of the sample was investigated using X-ray photoelectron spectroscopy (XPS, Thermo Scientific-MULTILAB 2000).

### 2.4. Electrode Fabrication and Electrochemical Measurements

**Scheme 1:**
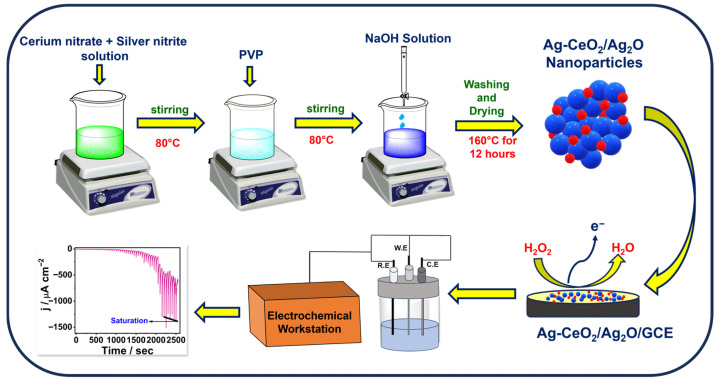
Schematic diagram of electrode fabrication process and electrocatalytic response of Ag-CeO_2_/Ag_2_O modified electrode towards the detection of H_2_O_2_.

Cyclic voltammetry and amperometry were used in electrochemical sensing experiments with a three-electrode electrochemical workstation. Before electrochemical analysis, the bare glassy carbon electrode (GCE) was cleaned with alumina slurry (0.3 µm). It was then thoroughly sonicated and washed with ethanol and deionized water to obtain a mirror-like surface. Next, added 5 mg of active electro-catalyst (Ag-CeO_2_/Ag_2_O nanostructure and CeO_2_ nanoparticles) to 1 mL of deionized water and sonicated for 2 h. Subsequently, 10 µL of the aforementioned suspension was dropped onto the GCE surface and allowed to dry at an ambient temperature. CeO_2_/GCE, and Ag-CeO_2_/Ag_2_O/GCE were used as working electrode, a saturated calomel electrode (SCE) was used as reference electrode, and platinum wire was used as counter electrode, respectively. Before the electrochemical experiments, the high-purity N_2_ gas was purged to remove the dissolved oxygen in the electrolyte solution. The cyclic voltammetry and amperometry were carried out at 0.1 M phosphate buffer solution. Scheme 1 shows the process of electrode modification with Ag-CeO_2_/Ag_2_O catalyst for electrochemical detection of H_2_O_2_ oxidation.

### 2.5. Real Sample Analysis

To examine the real sample analysis of CeO_2_ and Ag-CeO_2_/Ag_2_O/GCE modified electrodes towards H_2_O_2_ in tomato extracts spiked with the commercial antiseptic liquid. The above-mentioned extracts of 10, 20, and 30 µL were linearly added in 0.1 M PBS (pH 7.0) using cyclic voltammetry at 50 mV s^−^^1^. The recovery of spiked analyte was calculated for different concentrations.

## 3. Results

### 3.1. Structural, Morphological and Compositional Analysis

**Figure 1:**
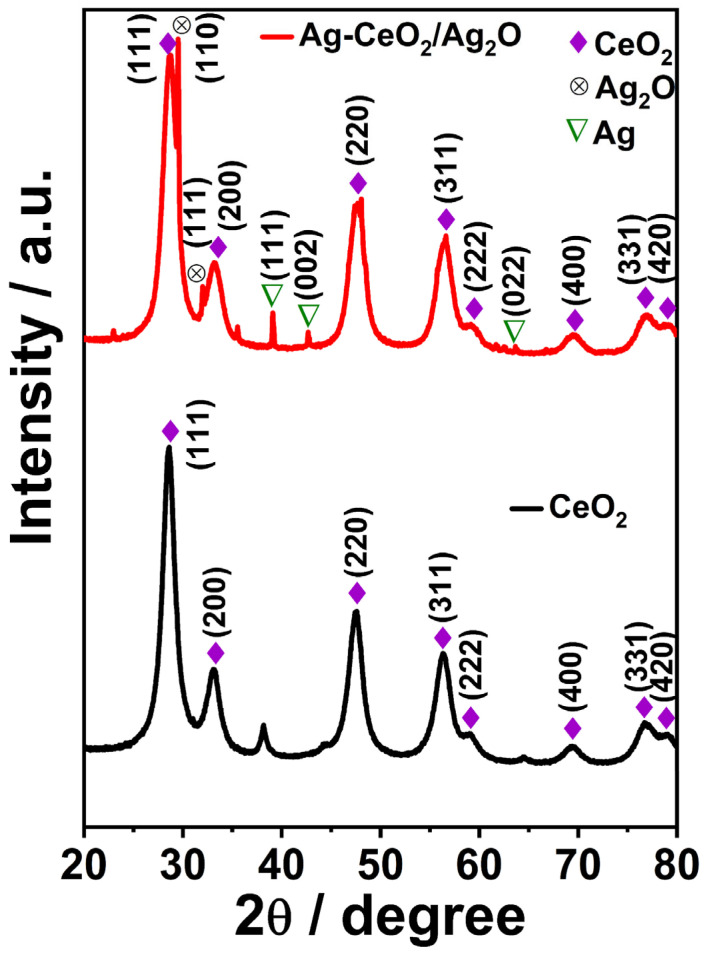
XRD pattern of the CeO_2_ and Ag-CeO_2_/Ag_2_O catalyst.

The crystalline nature of the synthesized CeO_2_ and Ag-CeO_2_/Ag_2_O nanocomposite is studied using X-ray diffraction (XRD), as shown in Figure 1. The XRD peaks appeared at 29.1°, 33.2°, 47.8°, 56.7°, 59.4°, 69.6°, 76.8°, and 78.9° corresponds to the (1 1 1), (2 0 0), (2 2 0), (3 1 1), (2 2 2), (4 0 0), (3 3 1), and (4 2 0) planes of cubic CeO_2_ crystal structure (JCPDS card No. 34–0394), respectively [30]. Also, the sharp diffraction peaks at 38.9°, 42.7°, and 63.8°, which can be indexed to the (1 1 1), (0 0 2), and (0 2 2) planes of the cubic structure of Ag, respectively (JCPDS card No. 04–0783) [31]. In addition, the minor peaks at 29.5° and 32.1° correspond to the (1 1 0) and (1 1 1) planes of the cubic structure of Ag_2_O, respectively (JCPDS card No. 43–0997) [21,31]. There is no additional peak presence in the sample, which is due to the high purity and high crystallinity of the sample. The sharp diffraction peaks show in the cubic structure of Ag doped along with the cubic structure of CeO_2_/Ag_2_O. Moreover, the higher intensity of the Ag-related diffraction peak is due to equal concentrations of the precursors. The sharp peaks arise due to some of the Ag ions decorated on the CeO_2_ surface. This result strongly implies that Ag nanoparticles and Ag-CeO_2_/Ag_2_O nanocomposite are present in the sample.

**Figure 2:**
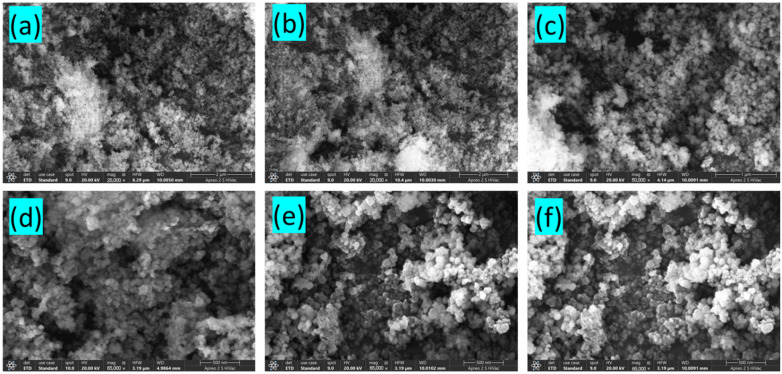
(**a**–**f**) FE-SEM images of the Ag-CeO_2_/Ag_2_O catalyst with different magnifications.

**Figure 3:**
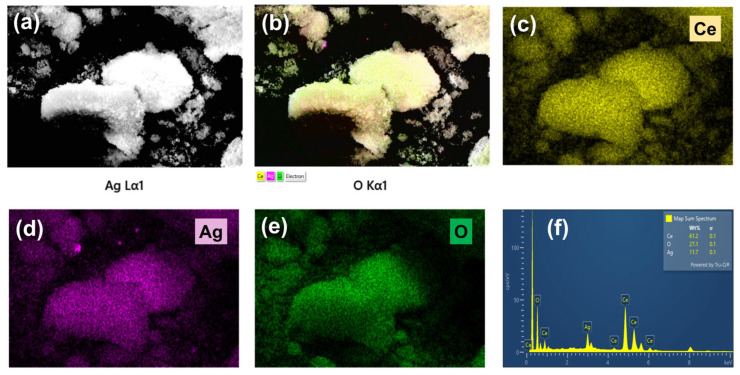
(**a**–**e**) EDS elemental mapping of the Ag-CeO_2_/Ag_2_O catalyst and (**f**) respective EDS spectrum of Ag-CeO_2_/Ag_2_O catalyst.

The microstructural properties of the Ag-CeO_2_/Ag_2_O catalyst were characterized using FE-SEM analysis, as shown in Figure 2. Figure 2a–f show the FE-SEM images at different magnifications clearly revealing that the nanosphere morphology has agglomerated tiny particles. Also, the EDS elemental mapping and corresponding EDS spectrum analysis of Ag-CeO_2_/Ag_2_O catalyst, as displayed in Figure 3a–f. It clearly demonstrates that Ce, Ag, and O elements are present in the sample.

**Figure 4:**
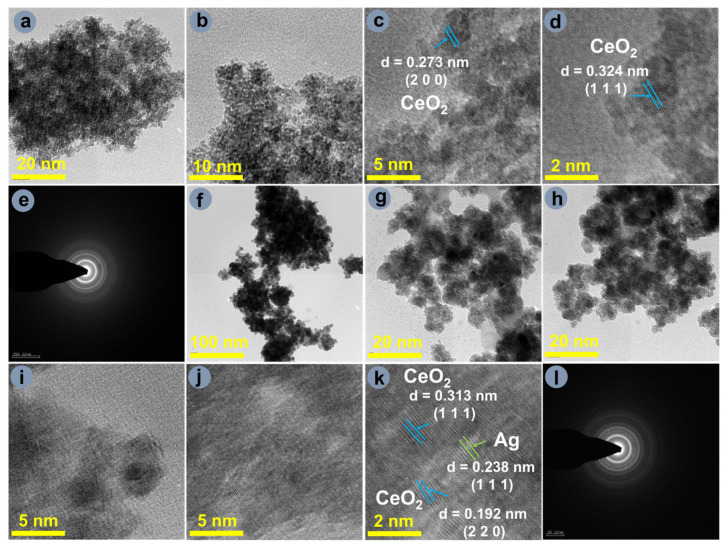
(**a**,**b**) Low-magnified HR–TEM images; (**c**,**d**) high-magnified HR–TEM images of CeO_2_ catalyst; and (**e**) the SAED pattern of the CeO_2_ catalyst. (**f**–**h**) Low-magnified HR–TEM images; (**i**–**k**) high-magnified HR–TEM images; and (**l**) the SAED pattern of the Ag-CeO_2_/Ag_2_O.

The morphological features of CeO_2_ and Ag-CeO_2_/Ag_2_O catalysts were analyzed via HR-TEM, and the images are shown in Figure 4. The HR-TEM images in Figure 4a,b show the agglomerated nanoparticles’ nanosphere-like morphology of the CeO_2_ catalyst, with the nanoparticles being homogeneously distributed. According to the same condition, the Ag-CeO_2_/Ag_2_O catalyst HR-TEM pictures were captured. Figure 4f–h shows the agglomerated nanoparticles and nanosphere-like morphology, and the Ag nanoparticles were uniformly dispersed on the CeO_2_ surface. The well-defined lattice fringes were estimated to be d = 0.324 nm and d = 0.273 nm, which correspond to the (1 1 1) and (2 0 0) planes of the CeO_2_ catalyst, respectively, in the high-magnification HR-TEM images of Figure 4c,d. On the other hand, the high-magnified HR-TEM images of Ag-CeO_2_/Ag_2_O catalyst (Figure 4i–k) lattice fringes were found to be d = 0.313 nm, and d = 0.192 nm corresponds to the (1 1 1), and (2 2 0) for CeO_2_, also the estimated d-spacing were about d = 0.238 nm corresponds to the (1 1 1) planes for Ag nanoparticles, respectively. The selected area electron diffraction (SAED) pattern of Figure 4e,l show the mono-crystalline of CeO_2_ catalyst and poly-crystalline nature of Ag-CeO_2_/Ag_2_O catalyst and high crystallinity nature. These HR-TEM results clearly reveal that Ag nanoparticles are incorporated into the CeO_2_ nanosphere lattice. Further, the homogeneous nanoparticles are exposed to the advantages of electrocatalytic behaviour because of the synergistic effect between Ag and CeO_2_/Ag_2_O nanoparticles. This structure cannot only benefit from advancing the electrochemical performance but also increase the electrocatalytic ability by easing the interfacial interaction between the electrode and the electrolyte. These HR-TEM images and SAED pattern implies improving the interfacial area between the Ag and CeO_2_/Ag_2_O nanoparticles, enhancing the electrochemical utilization of the electrochemical sensor.

**Figure 5:**
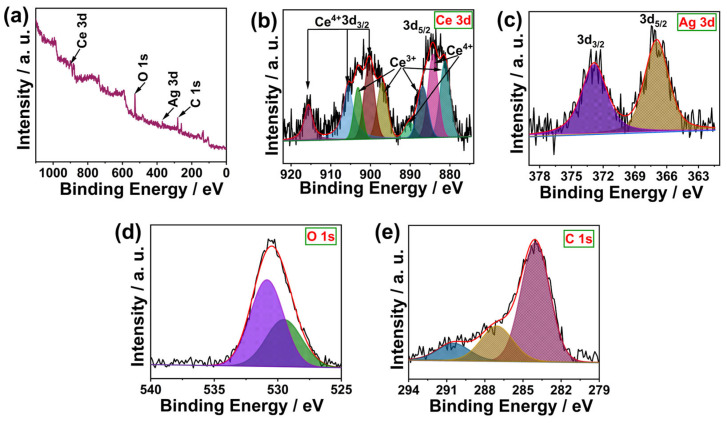
(**a**) XPS survey spectrum; (**b**,**c**) high-resolution Ce 3d spectrum, and Ag 3d spectrum, respectively. (**d**,**e**) High-resolution O 1s spectrum and C 1s spectrum, respectively, of the Ag-CeO_2_/Ag_2_O catalyst.

X-ray photoelectron spectroscopic investigation was conducted to investigate the chemical composition of Ag-CeO_2_/Ag_2_O, as illustrated in Figure 5. The XPS analysis confirms the presence of both metallic silver (Ag^0^) and ionic silver (Ag^+^), the latter predominantly in the form of Ag_2_O. The metallic Ag^0^ enhances electrical conductivity and provides additional catalytic active sites for efficient electron transfer. Meanwhile, Ag^+^ incorporation promotes the formation of oxygen vacancies within the CeO_2_ lattice, which play a key role in improving electrochemical sensing performance. During the sensing process, electrons from the electrode surface participate in electrochemical reduction reactions, and the synergistic effect of Ag^0^ and Ag^+^ significantly contributes to the enhanced sensitivity and stability of the sensor. Figure 5a shows the survey spectrum of Ce 3d, Ag 3d, O 1s, and C 1s. Figure 5b depicts the Ce 3d spectrum at 884.2 eV, and 900.4 eV corresponds to Ce 3d_5/2_ and Ce 3d_3/2_, respectively. Also, the corresponding satellite peaks at 881.2, 884.3, 886.8, 890.1 eV (3d_5/2_), also 897 eV, 900.1, 903.1, 905.6 eV (3d_3/2_), indicating the presence of Ce^3+/4+^ in the sample. In addition, the deconvolution peaks at 915.6 eV imply the presence of Ce^4+^ ions in the material [32]. The high-resolution XPS of Ag 3d spectra shown in Figure 5c shows the strong doublet peaks that correspond to Ag 3d_5/2_ and Ag 3d_3/2_ at energies of 366.9 eV and 372.8 eV, respectively, which implies that Ag^+^ is present in the sample [33]. As can be seen in Figure 5d, the sample’s surface and lattice oxygen display peaks at 529.4 eV and 530.8 eV in the high-resolution O 1s spectra. Peaks for C=O and C-O may be found in the high-resolution C 1s spectra of Figure 5e, which are at 284 eV, 287.1 eV, and 290.4 eV, respectively [34]. These XPS results revealed that all the elements and corresponding sample oxidation states of Ce^3+/4+^ and Ag^+^ ions are present in the sample.

**Figure 6:**
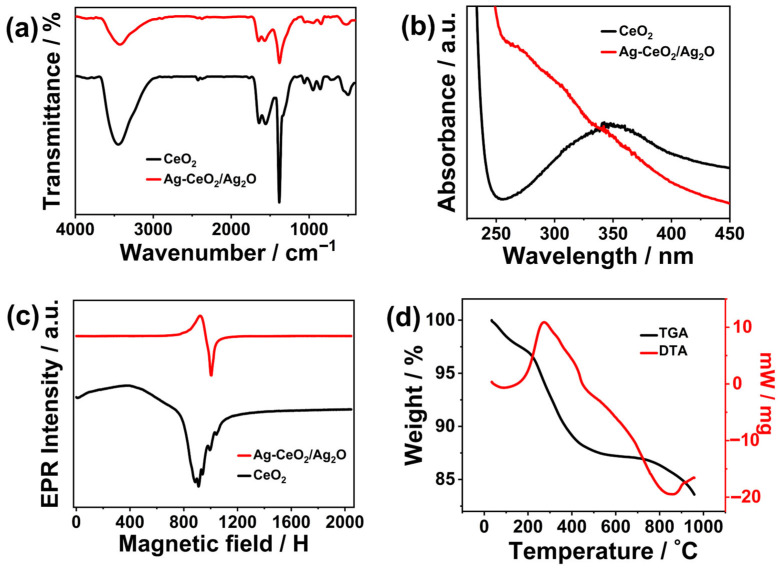
(**a**) FT-IR spectrum; (**b**) UV–visible spectrum; insert: overview of UV-vis spectrum; (**c**) EPR spectrum of CeO_2_ catalyst and Ag-CeO_2_/Ag_2_O catalyst; and (**d**) TGA-DTA curve of Ag-CeO_2_/Ag_2_O catalyst.

Figure 6a displayed FTIR spectra of CeO_2_ and Ag-CeO_2_/Ag_2_O. The vibrational peaks at 514.9 cm^−1^ are caused by the M–O bond and the metal–oxide formation [35]. Furthermore, bending vibrations of the O-H groups are detected at 1644.4 cm^−1^ and 3430.1 cm^−1^, which can be attributed to H_2_O molecules that have been adsorbed onto the sample surface. The strong absorption peaks at 1375.3 cm^−1^ are ascribed to the C–N stretching mode of the PVP monomer [36]. Figure 6b displays the UV–visible spectra of pure CeO_2_ and the Ag-CeO_2_/Ag_2_O nanocomposite. It clearly demonstrates that significant absorption peaks were discovered at 312.7 nm and 340.2 nm. Since Ag nanoparticles intercalate with the CeO_2_ surface lattice, their absorbance location changes away from the shorter wavelength area when compared to pure CeO_2_ due to the narrow band energy of the catalyst [37]. This optical property may be beneficial for enhancing the electrochemically active area of Ag-CeO_2_/Ag_2_O catalyst.

The magnetic properties of CeO_2_ and Ag-CeO_2_/Ag_2_O catalyst were studied by electron paramagnetic resonance (EPR) spectroscopic analysis. Figure 6c depicts an EPR signal displayed at 927.1 H due to the paramagnetic properties of the sample [38]. The EPR spectrum clearly shows that the intrinsic interaction between Ag^+^ ions and Ce^3+^ and Ce^4+^ cations results in the creation of Ag-CeO_2_/Ag_2_O catalyst [39].

The thermal stability of Ag-CeO_2_/Ag_2_O catalyst was examined by thermal gravimetric and differential thermal analysis (TG-DTA) spectrum as shown in Figure 6d. The TGA curve indicates two steps of weight-loss: The first loss was located at 85 °C, which is due to the removal of the excess reagent and water molecules. In addition, the second loss was placed at 205.6 °C due to the loss of organic agents present in the sample. Further, the DTA curve shows the strong exothermic peaks at 268 °C, which exhibit the improved crystallization of the nanoparticles. These TG-DTA results reveal the improvement of the crystalline nature of the Ag-CeO_2_/Ag_2_O catalyst.

### 3.2. Electrocatalytic Response of CeO_2_/GCE and Ag-CeO_2_/Ag_2_O/GCE Towards the Detection of H_2_O_2_

**Figure 7:**
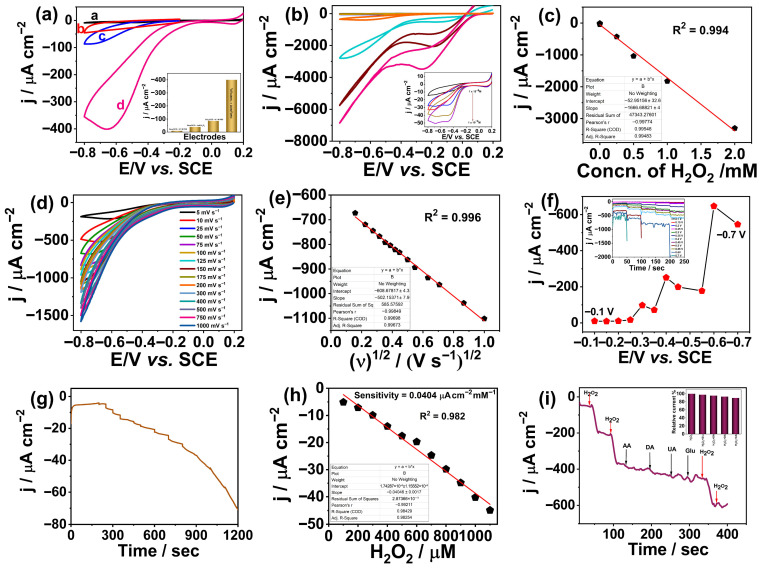
(**a**) CV response of different modified electrodes; insert: corresponding bar diagram; (**b**) CV response of CeO_2_/GCE at different concentration range from 0.1 to 2.0 mM; (**c**) corresponding calibration curve; (**d**) CV response of CeO_2_/GCE at different scan rates of 5–1000 mV s^−1^; (**e**) corresponding calibration curve of current density vs. (scan rate)^1/2^; (**f**) optimizing amperometry plot of CeO_2_/GCE at different potential window; inset amperometry response at different potential window from −0.1 to −0.7 V; (**g**) amperometry response of CeO_2_/GCE at −0.6 V towards H_2_O_2_ detection; (**h**) corresponding calibration plot at concentration ranging from 0.001 to 1 mM; (**i**) selectivity study of CeO_2_/GCE at different interferents; inset: corresponding bar graph of CeO_2_/GCE and respective calibration plot.

**Figure 8:**
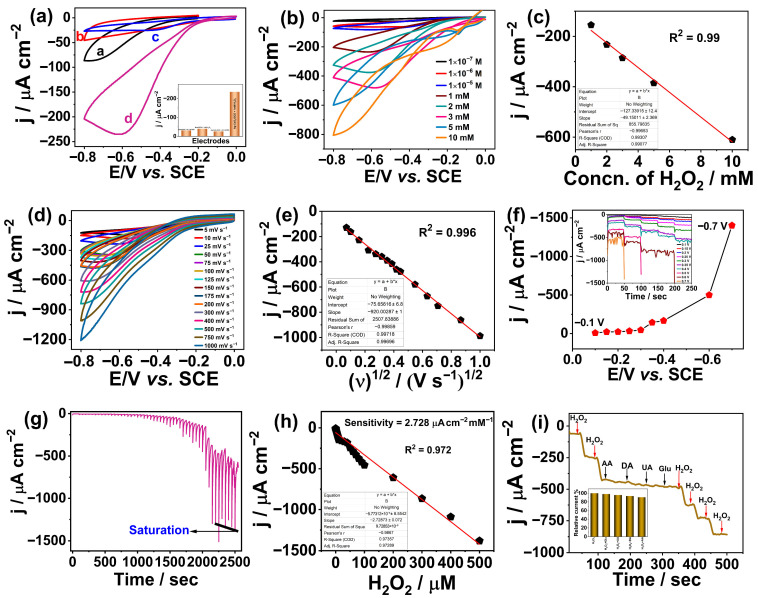
(**a**) CV response of different modified electrodes; insert: corresponding bar diagram; (**b**) CV response of Ag-CeO_2_/Ag_2_O/GCE at different concentration ranges, from 0.1 to 10 mM; (**c**) corresponding calibration curve; (**d**) CV response of Ag-CeO_2_/Ag_2_O/GCE at different scan rates of 5–1000 mV s^−1^; (**e**) corresponding calibration curve of current density vs. (scan rate)^1/2^; (**f**) optimizing amperometry plot of Ag-CeO_2_/Ag_2_O/GCE at a different potential window; inset: amperometry response at different potential window from −0.1 to −0.7 V; (**g**) amperometry response of Ag-CeO_2_/Ag_2_O/GCE at −0.6 V towards H_2_O_2_ detection; (**h**) corresponding calibration plot at concentration ranging from 10 nM to 0.5 mM; (**i**) selectivity study of Ag-CeO_2_/Ag_2_O/GCE at different interferents; insert: corresponding bar graph of Ag-CeO_2_/Ag_2_O/GCE and respective calibration plot.

Doping CeO_2_ with Ag nanoparticles significantly enhances its performance through multiple synergistic mechanisms. The incorporation of silver introduces additional active sites within the CeO_2_ lattice, accelerating the decomposition of H_2_O_2_ and thereby improving the peroxidase-like catalytic reaction that underpins the sensing mechanism. At the same time, Ag doping creates structural defects, particularly oxygen vacancies, which play a vital role in facilitating rapid electron and oxygen transfer, making the catalytic redox cycle more efficient and responsive. The presence of metallic Ag also improves the overall electrical conductivity of the CeO_2_ composite, ensuring stronger and more stable electrochemical signals and enabling the detection of H_2_O_2_ at lower concentrations. Furthermore, Ag doping modifies the morphology of CeO_2_ nanoparticles, resulting in smaller, more stable structures with higher surface-to-volume ratios that expose a greater number of catalytic sites to the analyte. Collectively, these effects lead to enhanced sensitivity, improved catalytic activity, and superior overall performance of Ag-doped CeO_2_ in sensing applications. Moreover, the presence of Ag_2_O along with Ag-doped CeO_2_ plays a synergistic role in enhancing H_2_O_2_ sensing performance. Ag_2_O, being a p-type semiconductor with excellent redox properties, facilitates additional oxygen vacancy formation and promotes rapid electron transfer during the catalytic decomposition of H_2_O_2_. Its coexistence with metallic Ag and CeO_2_ creates a heterojunction interface that improves charge separation and accelerates the redox cycle, thereby reducing recombination losses. Moreover, Ag_2_O provides supplementary catalytic sites and enhances the interaction between H_2_O_2_ molecules and the sensing surface, which boosts both sensitivity and response speed. Thus, the combined effects of Ag, Ag_2_O, and CeO_2_ establish a multi-functional catalytic network that delivers superior conductivity, higher catalytic efficiency, and improved overall sensor performance. The electrocatalytic behaviour of the suggested sensor in H_2_O_2_ electro-reduction was examined using CV techniques. The CVs of bare GCE, bare GCE with 1.0 mM H_2_O_2_, CeO_2_/GCE, and Ag-CeO_2_/Ag_2_O/GCE in 1.0 mM H_2_O_2_ with 0.1 M PBS (pH = 7.0) solution at 25 mVs^−1^ scan rate are displayed in Figure 7a and Figure 8a, respectively. The cyclic voltammogram demonstrates that no peak could be seen for the bare GCE. On the other hand, a poor decline response was observed at 1.0 mM H_2_O_2_. However, there is a redox peak seen at potentials of 0.6 V and 0.62 V for the modified CeO_2_/GCE and Ag-CeO_2_/Ag_2_O/GCE electrodes, respectively. The subsequent processes carry out the reduction of H_2_O_2_ and the associated oxidation [5].

H_2_O_2_ + e^−^ → OH + OH^−^ (1)

OH + e^−^ → OH^−^ (2)

2OH^−^ + 2H^+^ → 2H_2_O (3)

2H_2_O + 2e^−^ → 2H_2_O + O_2_ (4)

The mechanism of reduction of H_2_O_2_ for the modified electrode can be described by the following equations [5,7],

CeO_2_ + H_2_O_2_ ↔ Ce_2_O_3_ + O_2_ + H_2_O (5)

Ce_2_O_3_ + 2OH^−^ ↔ 2CeO_2_ + H_2_O_2_ + 2e^−^ (6)

Ag → Ag^+^ + 2e^−^ (7)

Ag^+^ → Ag^2+^ + e^−^ (8)

Ag^+^ + H_2_O_2_ → Ag^2+^ + HO• + OH^−^ (9)

Ag^2+^ + H_2_O_2_ → Ag^+^ + HO_2_• + H^+^ (10)

#### 3.2.1. H_2_O_2_ Detection by the Electrocatalytic Reaction of CeO_2_/GCE and Ag-CeO_2_/Ag_2_O/GCE

A study of the electrocatalytic behaviour of the developed sensor has been conducted using the exceptional electrochemical capabilities of CeO_2_/GCE and Ag-CeO_2_/Ag_2_O/GCE. Figure 7b and Figure 8b illustrate the cyclic voltammetry performance of CeO_2_/GCE and Ag-CeO_2_/Ag_2_O/GCE in 0.1 M PBS (pH 7.0) at 25 mV s^−1^ with increasing H_2_O_2_ concentrations. This indicates that the electrocatalytic behaviour of CeO_2_/GCE and Ag-CeO_2_/Ag_2_O/GCE towards the H_2_O_2_ sensor detection was enhanced since there was a simultaneous decrease in the peak current of anodic and a linear increase in the peak current at cathode with every addition of H_2_O_2_ (0.1–2.0 mM). The corresponding calibration plot of Figure 7c and Figure 8c shows the good correlation coefficient of R^2^ = 0.994 and R^2^ = 0.99, respectively.

#### 3.2.2. Effect of Scan Rate on CeO_2_/GCE and Ag-CeO_2_/Ag_2_O/GCE Towards the Detection of H_2_O_2_

As seen in Figure 7d and Figure 8d, the influence of scan rate on nano-CeO_2_/GCE and Ag-CeO_2_/Ag_2_O/GCE was carried out in the presence of 1 mM H_2_O_2_ in order to better understand the electrochemical reduction process of H_2_O_2_. At different scan rates between 5 and 1000 mV s^−1^, there was a noticeable enhancement of the peak current along with a shift in the cathodic peak towards the negative area observed for CeO_2_/GCE and Ag-CeO_2_/Ag_2_O/GCE. Furthermore, the linear relationship between the cathodic peaks and the square root of the scan rate vs. catalytic current (Figure 7e and Figure 8e) indicates that diffusion control is in place for the electrochemical mechanism regulating the reduction of H_2_O_2_ at Ag-CeO_2_/Ag_2_O/GCE. The respective correlation coefficients of R^2^ = 0.996 and R^2^ = 0.996.

#### 3.2.3. Amperometric Determination of H_2_O_2_ at CeO_2_/GCE and Ag-CeO_2_/Ag_2_O/GCE

We investigated the sensor’s behaviour under dynamic situations due to its improved Voltametric responsiveness. According to Figure 7f and Figure 8f and the related inset figures, the optimizing potential window for H_2_O_2_ sensor detection suggests that 0.6 to −0.6 V is appropriate for additional amperometric determination. Consequently, as seen in Figure 7g and Figure 8g, amperometry sensor measurements for CeO_2_/GCE and Ag-CeO_2_/Ag_2_O/GCE were performed under ideal circumstances with successive additions of H_2_O_2_ at an operating potential of 0.6 V into the constantly stirring saturated 0.1 M PBS. With each H_2_O_2_ injection, the catalytic current increased rapidly. However, given the same concentrations, CeO_2_/GCE showed a lower current response. The calibration curve for catalytic currents as a function of H_2_O_2_ concentration for CeO_2_/GCE and Ag-CeO_2_/Ag_2_O/GCE is displayed in Figure 7h and Figure 8h. The results showed that the sensitivity for CeO_2_/GCE was 0.0404 µA cm^−2^ µM^−1^ (100 µM–1 mM) and that the sensitivity for Ag-CeO_2_/Ag_2_O/GCE was 2.728 µA cm^−2^ µM^−1^ (10 nM–0.5 mM) with corresponding limits of detection (LOD) and limits of quantification (LOQ) of 129.3 µM and 6.34 µM as well as 431.1 µM and 21.16 µM, respectively. The sensitivity was calculated by dividing the area of the working electrode (0.196 cm^2^) by the slope (m) of the calibration plot. The associated correlation coefficients were R^2^ = 0.982 and 0.972, respectively. When the constructed sensor’s performance was compared to that of a previously published H_2_O_2_ electrochemical sensor, it became clear that the fabricated sensor performed significantly better, as shown in Table 1. The enhanced electrochemical properties of Ag-CeO_2_/Ag_2_O/GCE, which were attained by fortifying the electron transfer mechanism, are responsible for the remarkable efficacy of the electrochemical sensor. The electrochemical breakdown of H_2_O_2_ led to an increase in catalytic current and a decrease in detection potential.

#### 3.2.4. Effect of Interfering Studies of CeO_2_/GCE and Ag-CeO_2_/Ag_2_O/GCE

Anti-interference performance is one of the essential parameters for an electrochemical sensor to explore its practical applicability. Consequently, the amperometry performance of CeO_2_/GCE and Ag-CeO_2_/Ag_2_O/GCE towards the detection of H_2_O_2_ under constant stirring conditions. The selectivity study of CeO_2_/GCE and Ag-CeO_2_/Ag_2_O/GCE for the measurement of H_2_O_2_ is displayed in Figure 7i and Figure 8i, together with four excess agents of interferents, including ascorbic acid (AA), dopamine (DA), uric acid (UA), and glucose (Glu). From the corresponding bar chart diagram of insert Figure 7i and Figure 8i, the improved sensor revealed that there was no current signal for the other interferences, only a catalytic current response when H_2_O_2_ was added, when the concentration of interfering molecules is three times higher than that of H_2_O_2_. From the analysis, the sensitivity of the modified electrode towards H_2_O_2_ in the presence and absence of interfering molecules. Inserts of Figure 7i and Figure 8i demonstrate the retention in sensitivity of 97.6%, 95.3%, 92.8%, and 89.5% for CeO_2_/GCE and 98.2%, 95.9%, 93.6%, and 90.8% for Ag-CeO_2_/Ag_2_O/GCE, respectively. In light of this, the newly designed Ag-CeO_2_/Ag_2_O/GCE sensor provides a high level of selectivity for H_2_O_2_ measurement.

#### 3.2.5. Stability, Repeatability, and Reproducibility of CeO_2_/GCE and Ag-CeO_2_/Ag_2_O/GCE

**Figure 9:**
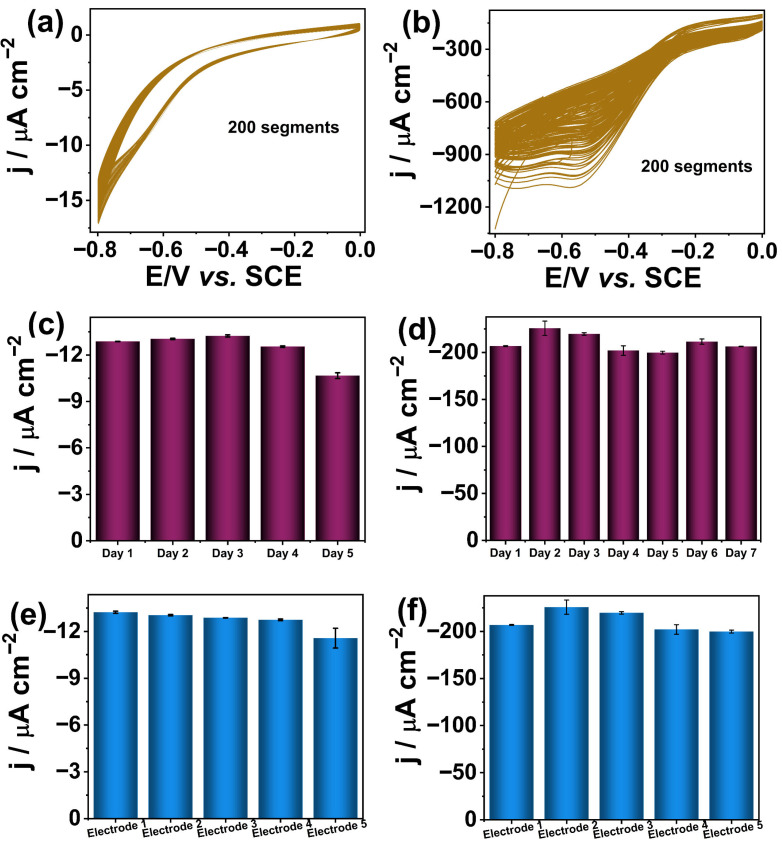
(**a**,**b**) CV response of CeO_2_/GCE and Ag-CeO_2_/Ag_2_O/GCE in the presence of 100 µM H_2_O_2_ for 200 cycles at 50 mV s^−1^; (**c**,**d**) bar graph of storage stability; (**e**,**f**) reproducibility bar graph of CeO_2_/GCE and Ag-CeO_2_/Ag_2_O/GCE, respectively.

The repeatability, reproducibility, and stability performance are other crucial parameters in the proposed sensor. Figure 9a,b show the cyclic stability of CeO_2_ and Ag-CeO_2_/Ag_2_O modified electrodes, which were subjected to 200 continuous cycles using CV at 50 mV s^−1^ in the presence of 100 µM H_2_O_2_. The result demonstrates that from the first cycle to the 200th cycle, there is no significant deviation, revealing good repeatability. The storage stability studies were examined for both CeO_2_ and Ag-CeO_2_/Ag_2_O/GCE electrode in the presence of 100 µM H_2_O_2_ in 0.1 M PBS (pH 7.0) at 50 mV s^−1^ every day, and the results were plotted as a bar graph with error bar as shown in Figure 9c,d. On the first day, the current value of 206.8 µA was observed, and on the seventh day, the current value was 206 µA. These results imply that the seventh day of current value reduction is negligible, suggesting that our modified electrode maintains excellent stability performance.

Further, the reproducibility studies were employed using five different electrodes that were separately modified with CeO_2_ and Ag-CeO_2_/Ag_2_O/GCE. Their response towards 100 µM H_2_O_2_ added in 0.1 M PBS (pH 7.0) at 50 mV s^−1^ was studied, and its internal repeatability was also studied by measuring the CV at 5 min intervals for each electrode and plotted as a bar graph with error bars, as shown in Figure 9e,f. It has been displayed that all the modified electrodes exhibit similar current responses for the detection of H_2_O_2_. All the studies suggest that the Ag-CeO_2_/Ag_2_O-modified electrode possesses an appropriate and efficient electrode for a developed sensor.

#### 3.2.6. Real Sample Analysis of CeO_2_/GCE and Ag-CeO_2_/Ag_2_O/GCE

Real sample analysis is a significant factor in practical applications. For the real sample analysis of H_2_O_2_ in tomato extracts, the 1, 2, and 3 µM of commercial antiseptic liquid was spiked into the tomato extract and linearly added in 0.1 M PBS (pH 7.0) at 50 mV s^−1^ in both CeO_2_ and Ag-CeO_2_/Ag_2_O modified electrodes, respectively. Table 2 and Table 3 show that good recovery of 96.8–108% (Tomato extract), also 100–100.7% (Medical antiseptic), and 101.1–104.5% (Tomato extract), also 97.3–100.3% (Medical antiseptic), respectively. This real sample analysis demonstrates that our fabricated electrode is a promising and effective electrode for the proposed H_2_O_2_ sensor.

## 4. Conclusions

In this study, a simple and efficient chemical synthesis route was employed to prepare a silver-doped cerium oxide/silver oxide (Ag-CeO_2_/Ag_2_O) nanocatalyst. The synthesized material was comprehensively characterized using various physicochemical techniques, including XRD, FE-SEM, HR-TEM, FT-IR, UV–Vis spectroscopy, EPR, TG-DTA, and XPS. The Ag-CeO_2_/Ag_2_O nanocomposite was subsequently used to fabricate a modified glassy carbon electrode (Ag-CeO_2_/Ag_2_O/GCE) via a drop-casting method for the electrochemical detection of hydrogen peroxide (H_2_O_2_). The sensing performance of both CeO_2_/GCE and Ag-CeO_2_/Ag_2_O/GCE was evaluated. The coexistence of metallic Ag^0^ and ionic Ag^+^ in Ag–CeO_2_ enhanced both conductivity and catalytic activity. Ag^+^ incorporation generated oxygen vacancies, which further boosted electron transfer and redox processes. Together, these effects significantly improved the sensitivity and stability of hydrogen peroxide sensing. The Ag-doped sensor exhibited significantly enhanced sensitivity (2.728 µA cm^−^^2^ µM^−1^) compared to the undoped CeO_2_ sensor (0.0404 µA cm^−^^2^ µM^−1^). It also demonstrated a broad linear detection range from 10 nM to 0.5 mM, with a remarkably low limit of detection (LOD) and limit of quantification (LOQ) of 6.34 µM and 21.1 µM, respectively. In contrast, CeO_2_/GCE showed LOD and LOQ values of 129.3 µM and 431.1 µM. Selectivity studies revealed minimal interference from common coexisting analytes, highlighting the excellent selectivity of the Ag-CeO_2_/Ag_2_O-modified electrode. Moreover, the sensor demonstrated outstanding repeatability, reproducibility, and long-term operational stability. The high recovery rate in real sample analysis further confirms the practical applicability of the proposed non-enzymatic sensor for precise and reliable H_2_O_2_ detection.

## Figures and Tables

**Table 1:** Comparisons of performances of different electrochemical sensors for H_2_O_2_.

S. No.	Electrode Material	Method	E_p_ (V)	Linear Range (M)	Sensitivity (μA mM^−1^)	LOD (M)	Ref.
1	Fe_3_O_4_@MoS_2_-Au/GCE	SWV	−0.7–0.1 V	1 × 10^−6^ to 120 × 10^−6^	-	10^9^ × 10^−9^	[40]
2	MoO_3_/Ni-F/GCE	LSV	−1–+1 V	0.072 × 10^−6^ to 74.85 × 10^−6^	-	1.2 × 10^−6^	[41]
3	GCE/poly(CoTBImPc)	CA	−0.45 V	10 × 10^−9^ to 100 × 10^−9^	0.2482 µA µM^−1^ cm^−2^	3.0 × 10^−9^	[42]
4	GCE/CNT/poly(CoTBImPc)	CA	−0.45 V	10 × 10^−9^ to 100 × 10^−9^	3.4522 mA nM^−1^ cm^−2^	2.0 × 10^−9^	[42]
5	Poly(CuPc)/MGE	CV	0–−0.7 V	0.35 × 10^−6^ to 70 × 10^−6^	-	0.25 × 10^−6^	[43]
6	GCE/poly(CoTBIPc)	CA	−0.55 V	3 × 10^−6^ to 140 × 10^−6^	-	0.8 × 10^−6^	[44]
7	GCE/GO/poly(CoTBIPc)	CA	−0.55 V	2 × 10^−6^ to 200 × 10^−6^	-	0.6 × 10^−6^	[44]
8	CeO_2_/GCE	CA	−0.6 V	100 × 10^−6^ to 1.0 × 10^−3^	0.0404	129.3 × 10^−6^	This work
9	Ag-CeO_2_/Ag_2_O/GCE	CA	−0.6 V	10 × 10^−9^ to 0.5 × 10^−3^	2.728	6.34 × 10^−6^	This work

**Table 2:** H_2_O_2_ detection in real samples, such as commercial antiseptics and spiked tomato samples, using CeO_2_/GCE modified electrode.

Samples	Detected (μA)	[H_2_O_2_] Added (μM)	Found (μA)	Recovery (%) (n = 3)
Medical Antiseptic	−15.36 −22.57 −36.80	500 800 1100	−14.68 −22.70 −36.80	104.79 99.11 100
Tomato	−18.20 −27.61 −47.68	700 900 1100	−18.80 −26.76 −45.75	96.8 103.37 104.15

**Table 3:** H_2_O_2_ detection in real samples, such as commercial antiseptics and spiked tomato samples, using an Ag-CeO_2_/Ag_2_O/GCE modified electrode.

Samples	Detected (μA)	[H_2_O_2_] Added (μM)	Found (μA)	Recovery (%) (n = 3)
Medical Antiseptic	−94.03 −146.50 −237.82	5 9 13	−96.57 −146.07 −243.78	97.36 100.31 97.55
Tomato	−32.46 −33.30 −36.35	0.8 0.8 0.9	−32.10 −32.10 −34.78	101.12 103.73 104.51

## Data Availability

The dataset is available on request from the authors.
